# Primary care physicians' experience of caring for children with parents with mental health illness: a qualitative study among French general practitioners and paediatricians

**DOI:** 10.1186/s12875-023-02145-y

**Published:** 2023-09-18

**Authors:** Cécile Ribette, Lucie Rosenthal, Jean-Philippe Raynaud, Ludivine Franchitto, Alexis Revet

**Affiliations:** 1 Department of Child and Adolescent Psychiatry, Toulouse University Hospital, Toulouse, France; 2 CERPOP, University of Toulouse, Inserm, UPS, Toulouse, France

**Keywords:** Parental psychiatric disorders, Attachment disorder, Private physicians’ experiences, Qualitative research, Interpretative phenomenological analysis

## Abstract

**Background:**

Parental psychiatric disorders can have a significant impact on child development and the parent-infant bond, with a high risk of attachment disorders. Early identification of difficulties in the parent–child relationship is essential to prevent consequences for the child. Childcare practitioners have a major role to play in this early detection process, through regular mandatory consultations during the first two years of a child's life. Thus, the aim of this study was to collect the experience of private practitioners in their care of children of parents with a mental health illness.

**Method:**

This is a cross-sectional, observational, qualitative study. Data were collected by means of semi-structured interviews with eleven general practitioners and private paediatricians between February and July 2021 in Toulouse and its suburbs. We only included practitioners who had followed children of parents with a mental health illness. The interviews were recorded with the agreement of the participants, before being transcribed anonymously. The data were analysed with NVivo software using interpretative phenomenological analysis.

**Results:**

Three main themes emerged from the results, which were further divided into several sub-themes. Addressing psychiatric disorders presents a risk for the therapeutic relationship. Practitioners express a need to preserve this relationship with the parent in joint care. Care is difficult and is permeated by the parents' emotional issues. Furthermore, practitioners face a conflict between their concerns for the parent–child bond and their desire not to stigmatise these families. They express a feeling of isolation in these follow-ups. This stressful care has a significant emotional impact on the doctors. Access to psychiatric training and multidisciplinary collaboration seem to be essential to improve the follow-up experience for practitioners, as these factors strengthen inter-professional connections.

**Conclusion:**

Practitioners describe a parent-doctor relationship at risk, which is underpinned by the fear of care placement. This study illustrates the need to strengthen multidisciplinary work by promoting interprofessional exchanges, in order to improve the experience of practitioners in this care process. Addressing practitioners’ fear of discussing parental psychiatric illness is very important, so as not to delay the implementation of preventive actions that are likely to improve the developmental prognosis for children.

## Background

Improvement of mental health care has enabled people suffering from mental disorders to better invest their parenthood, due to less recourse to isolation in specialised institutes and to a better tolerance profile of psychotropic drugs [1]. For example, 20–60% of those who receive mental health care in Australia and the United States are parents [1–4]. In addition, 10–20% of women are thought to develop a psychiatric disorder during pregnancy or in the first post-partum year, more than 1% of whom develop severe disorders (schizophrenia spectrum disorders, bipolar affective disorders and/or severe depression) [5].

A large body of research has investigated the potential impact of these parental disorders on child development [6]. A literature review published in 2017 found a potential impact on psychomotor, language and behavioural development in children. An impact on the parent–child relationship, with an effect on attachment was also identified [7]. Parents who suffer from mental illnesses must reconcile parenthood with the management of their disorder, which can make them unavailable from time to time, resulting in parental discontinuity, both physically and psychologically [7, 8]. Mothers with schizophrenia may have unsettling or poor interactions with their children [9, 10]. In addition, maternal antenatal depression is associated with an increased risk of disorganised attachment in children at 12 months of age [11]. These attachment disorders in turn have repercussions on the child's development and interpersonal relationships, which may persist into adulthood [7–12]. Studies also identify an association between these disorders and physical comorbidity (chronic pain, cardiovascular diseases and inflammatory pathologies) [11, 13].

Early identification of these attachment difficulties is essential for early intervention and parenting support in order to prevent an impact on the child [7, 14, 15]. Early childhood practitioners provide first-line care and play a key role in identifying children at risk through regular mandatory appointments during the first two years of life, a crucial period for attachment formation [8]. Furthermore, parents with mental illnesses seek out the parenting support role that these practitioners can provide [16, 17].

There are few studies on the experience of healthcare professionals concerning the parenting of patients with psychiatric disorders. Among psychiatrists who care for adults, the main results reveal fear of a breakdown in the therapeutic relationship with the patient if parenting is addressed, a feeling of incompetence, and difficulty coordinating with child and adolescent psychiatric services [16, 18, 19]. However, to the best of our knowledge, there are no studies that focus on the experiences of early childhood practitioners on this subject.

Consequently, we decided to focus on the experiences of these private practitioners in the care of children whose parents suffer from psychiatric disorders. We conducted an exploratory qualitative study, using semi-structured interviews. The main objective of this research was to record the experience of professionals in these follow-ups, in order to identify their difficulties and needs. The aim of this work is to highlight areas that require improvement and strengthen the coordination of the multidisciplinary network in private practice.

## Materials and methods

The study focused on French general practitioners and paediatricians working in private practice with children in their care whose parents suffer from psychiatric disorders. The interviewer and data interpreters are psychiatrist specialised in child and adolescent psychiatry. Their interests in this topic came from their clinical experience especially in perinatal psychiatric care, where interdisciplinary exchange is essential. We were aware of the importance of partnership work and support difficulties encountered by practitioners in these situations. The aim was to explore the experiences of professionals and to propose ways of improving the coordination of care.

The participants were informed about the research and the opportunity to participate through a mail shared with the associations of private paediatricians and of general practitioners in the area, with the permission of the heads of these associations. They were then directly contacted by the investigator via e-mail or telephone to offer to participate in the study. The condition for inclusion in the study was that they had experience in care for children whose parents had a psychiatric disorder. Recruitment ran from February to July 2021 with the inclusion of eleven professionals aged 31 to 75 years. The main socio-professional characteristics of the doctors interviewed in Toulouse and its region are summarised in Table 1. Although we tried to reach as many men as women, more female practitioners were included which may be partly due to the increasing proportion of women working in the medical field in France [20]. Some practitioners declined to participate due to lack of availability or lack of patients in their practice who matched the topic of the study. Participants were not related in any way to the investigators. Some were able to put us in touch with other colleagues who might be interested in our study, thus creating a network of interest around the issue at hand.

**Table 1:** Demographic and professional characteristics of healthcare professionals

Practitioner number	Speciality	Gender	Age (years)	Experience in private practice (years)
P1	Paediatrician	Female	53	25
P2	Paediatrician	Female	56	25
P3	General Practitioner	Male	63	15
P4	General Practitioner	Female	63	37
P5	General Practitioner	Female	58	15
P6	Paediatrician	Male	61	31
P7	General Practitioner	Female	33	4.5
P8	Paediatrician	Female	46	6
P9	General Practitioner	Female	31	2
P10	Paediatrician	Female	32	4
P11	General Practitioner	Male	71	44

Data were collected by means of semi-structured interviews of approximately 30 to 75 min, according to an interview grid presented in Table 2. The interviews were conducted in French with secondary translation into English after analysis of the results.

**Table 2:** Interview grid

Areas assessed	Type of questions
Career path	What is your profession and how long have you been in private practice?
Clinical experience	In your practice, have you ever had to follow children with parents who suffer from psychiatric disorders? What was it like? Can you think of a specific situation? What made you aware?
Feelings about the care of these families:	Did you find it complicated? If so, how? How did you handle the parent’s reactions in these situations?
Needs related to support	What are your needs in these situations?
Impact on development	Have you ever had particular concerns about a child's development because of the parent’s illness?
Parent–child relationship if not previously mentioned by the practitioner	Have you ever had specific concerns about the bond between a child and parent due to the parent’s illness?

These individual interviews were conducted by the principal investigator (PI) at the practitioner's office or home. The interviewees' anonymous comments were recorded with their consent and then transcribed. The data were analysed by Phenomenological Interpretative Analysis (IPA) [21]. The interviews were coded using NVivo 12 Plus software [22]. The themes and meta-themes were validated by two senior child psychiatrists (LF and LR) who supervised the research, allowing triangulation of the coding. Data saturation was reached at interview 9, as no additional themes emerged and the narratives were similar in the final interviews.

This study meets the COREQ criteria for validation of a qualitative study [23]. The data were processed according to the CNIL reference methodology MR-004 [24]. In compliance with the General Data Protection Regulation, the research was registered in the Toulouse University Hospital internal register under the reference RnIPH 2021–26.

## Results

### Addressing parental psychiatric disorders: a risk for the relationship

#### Practitioners' views of mental illness: blurred lines

Doctors are hesitant and uncertain about naming psychiatric symptoms and disorders. They may question whether incest, drug addiction or social insecurity fall within the field of mental illness. The question of whether “anxiety-depressive” disorders fall within the realm of psychiatry was raised several times by the doctors who were interviewed, in contrast to more clearly identified illnesses such as schizophrenia and bipolar disorder, as if their frequency in the general population and their more common nature would make these disorders a matter for general medicine thus questioning an overlap of competences between practitioners.

“I'm going to exaggerate when I say that, I think we're all mentally ill: [mimics a length scale, and brings his hand back at different intervals]: just a little bit, a little bit more, really a lot" (P3).

#### A delicate subject to address

The majority find it difficult to ask about parental history of mental illness, which requires a special effort.

“Now, I try to ask about psychiatric illnesses in the history, but it's still not easy” (P2).

Therefore, background information is often collected in a roundabout way, sometimes through a third party, such as a grandparent. These barriers lead to a delay in the identification of mental illnesses in parents.

« Moreover, I had been alerted by the mother's mother, who told me that her daughter was bipolar» (P9).

#### Preserving the relationship: a challenge for practitioners

The majority of the practitioners who were interviewed stressed the importance of the therapeutic relationship with the parent, an essential third party in the child’s care. Most practitioners approach the subject indirectly, for example by relying on the fact that certain disorders are now considered normal, such as post-partum depression. Others rely on identification by sharing personal experiences.

“Because as soon as we feel that things are not going well, we use the fact that baby blues are now considered normal or post-partum depression, which is quite common” (P1).

These strategies are not always effective, and the risk of disruption of care is ever present. One physician recounted losing contact with a child after trying to formulate a description of the maternal characteristics without stigmatisation*.* Therefore, on a daily basis, practitioners are confronted with this complex relationship with the parents of the children they care for.

### Stressful care

#### A therapeutic relationship permeated by the parents' emotional issues

Physicians can be the target of attacks and the receptacles of significant displaced anxiety. One physician described the aggressiveness of a father in response to her refusal to prescribe a sedative for his baby, whose crying at night he could no longer tolerate.

“I was exhausted, so exhausted, then I felt attacked because I could feel his anger, his face was red behind the mask [...], sometimes it drives me to despair” (P2).

The fear of care placement is often identified as the underlying cause for these outbursts. Addressing psychiatric problems quickly arouses the fear in parents of being reported or even separated from their child.

“It's difficult, I try to use the child's welfare as a reason, or to validate them, [...] the parents, to build their confidence because I don’t want to make them suffer, but often they retreat” (P4).

#### Paediatric consultations marked by anxiety

This relationship with the parent causes physicians to adapt their approach both in the exercise of their profession and in their way of thinking about care. Some shared their preconceptions about the possible consequences of the parent's mental illness on the children. There is a contrast between an expressed desire not to stigmatise these families and the concern about the impact of the parents’ illness on the parent–child bond or on the child's development. Most physicians are particularly vigilant about parent–child interactions, including observing dressing times during consultations.

“I was very afraid of that, really that the connection would not be made, that they would not be able to form that bond [...] because of their illness, so yes it was a major worry, totally” (P9).

#### An emotional impact on practitioners

All the physicians who were interviewed emphasised how very isolated they felt providing this type of care. One even went so far as to draw a comparison with humanitarian medicine.

“I'll draw a parallel [:] apart from being a general practitioner, [I practised] humanitarian medicine, where you are all alone in the middle of nowhere. Well, you get out the knife and the string, and get on with it” (P3).

This reality is accompanied by a sense of guilt, either that there is nothing more that can be done for the child or the parent, or that something is being missed. An experience of powerlessness is also frequently expressed in these follow-ups. Some practitioners confided to us that they would like to have a change in referral doctor, which would “*relieve*” them of these stressful follow-ups.

Therefore, these follow-ups are impacted by the fear of harming the child or his/her family. Professionals are also affected by the fear of care placement and the fear of causing it. Others, on the contrary, may feel that their approach prevents a separation that is necessary.

"I was the only one [...] who managed to prevent this mother from being separated from her children" (P3).

### What are the options in this type of care?

#### Personal and external resources

Several of the practitioners who were interviewed admitted to feeling incompetent in these follow-ups, due to their lack of knowledge of psychiatric disorders. A recently established private practitioner questioned her ability to follow an infant whose mother was diagnosed as bipolar and whose father suffered from a depressive disorder. The issue of mental health training is central. Physicians who have had specialised theoretical training say that they feel more confident with this type of care.

On the other hand, physicians identify different resources that are available when a referral is needed. They rely on the adolescent network, which is considered to be “*extremely valuable for all adolescents” (P5)*, and the perinatal psychiatric institutions. These facilities are identified as services that “*can be relied on” (P2),* enabling them to jointly refer the parent and the child to a care unit*.* However, problems are reported in referrals for children with psychiatric disorders, regardless of the mental status of the parents. Physicians often have difficulty understanding the organisation of the child psychiatric care system, including the specific features of the different institutions.

“What is complicated [...] is always the muddle of different institutions, private practice, […], 30 years of practice and I still don’t understand the difference between CMP*, CMPP**, ATTP***, day hospital, guidance centres, etc” (P6).

*CMP (Medical-psychological Centre), **CMPP (*Medico-psycho-educational Centre*), ***ATTP (*Part-time Therapeutic Facility*) which are public psychiatric outpatient facilities.

**Fig. 1:**
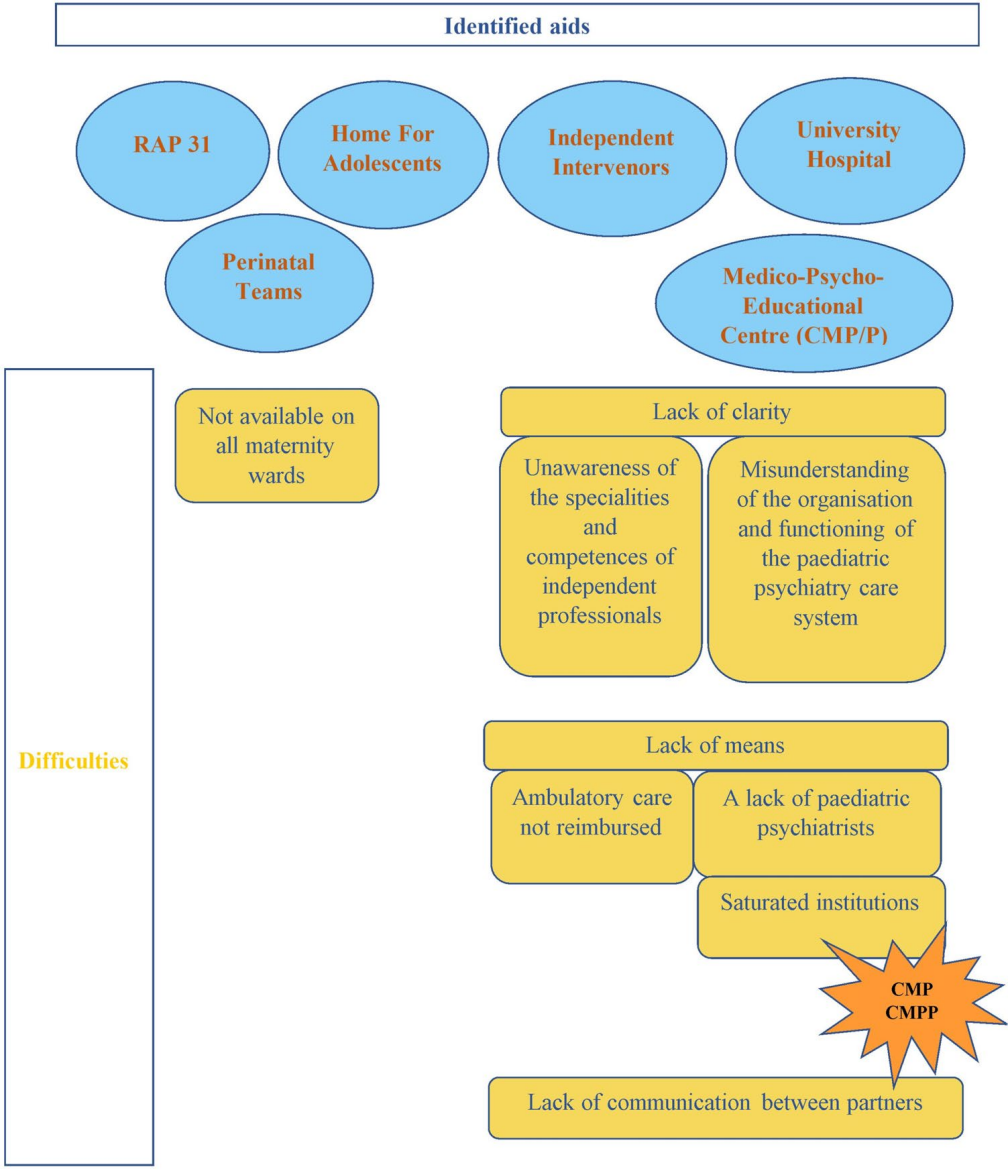
Representations of the resources identified and the difficulties encountered by the practitioners. **CMP / CMPP:** Medico-Psychological Centre, Medico-Psycho-Educational Centre. **RAP31**: Adolescence Network Partnership 31

The saturation of institutions, particularly CMPs and CMPPs, causes significant delays in treatment, and difficulty referring children early. These difficulties are accompanied by an acute awareness that the resources allocated to child psychiatry are insufficient. These barriers can end up discouraging physicians from referring children to public health care institutions: “*The CMPs are inaccessible, so I don't even send them to there, it's not even worth it” [laughs] (P5).* Outpatient psychological care is also limited by the fact that it must be paid for, which many families cannot afford. Figure 1 summarises the resources identified and the difficulties noted in their use (Fig. 1).

#### Connections: an essential factor for this type of support

All the participants complained of a lack of connection and coordination with partners*,* despite the observed benefit of these exchanges, which provide support in these “*difficulty situations” (P8)* and help practitioners to adapt their approach, through a better understanding.

“When we manage to make a connection, and we have the child psychiatry referral, and the child psychiatrist communicates with us, we understand the situation better and we are able to find a better approach [...] it is extremely beneficial to the patient and the practitioner*”* *(P2).*

All the physicians who were interviewed expressed the wish to work in a multidisciplinary manner, “*to form an alliance with a group of professionals, to be able to support this family” (P11).* Some doctors would like to have joint consultations with mental health professionals. The vast majority of practitioners suggest the creation of a consultation and referral network to provide guidance for professionals. According to them, this would make it possible to centralise difficult cases, or those in diagnostic limbo. There were also calls for centralisation of psychologists and their specialisations, in order to facilitate referrals.

**Fig. 2:**
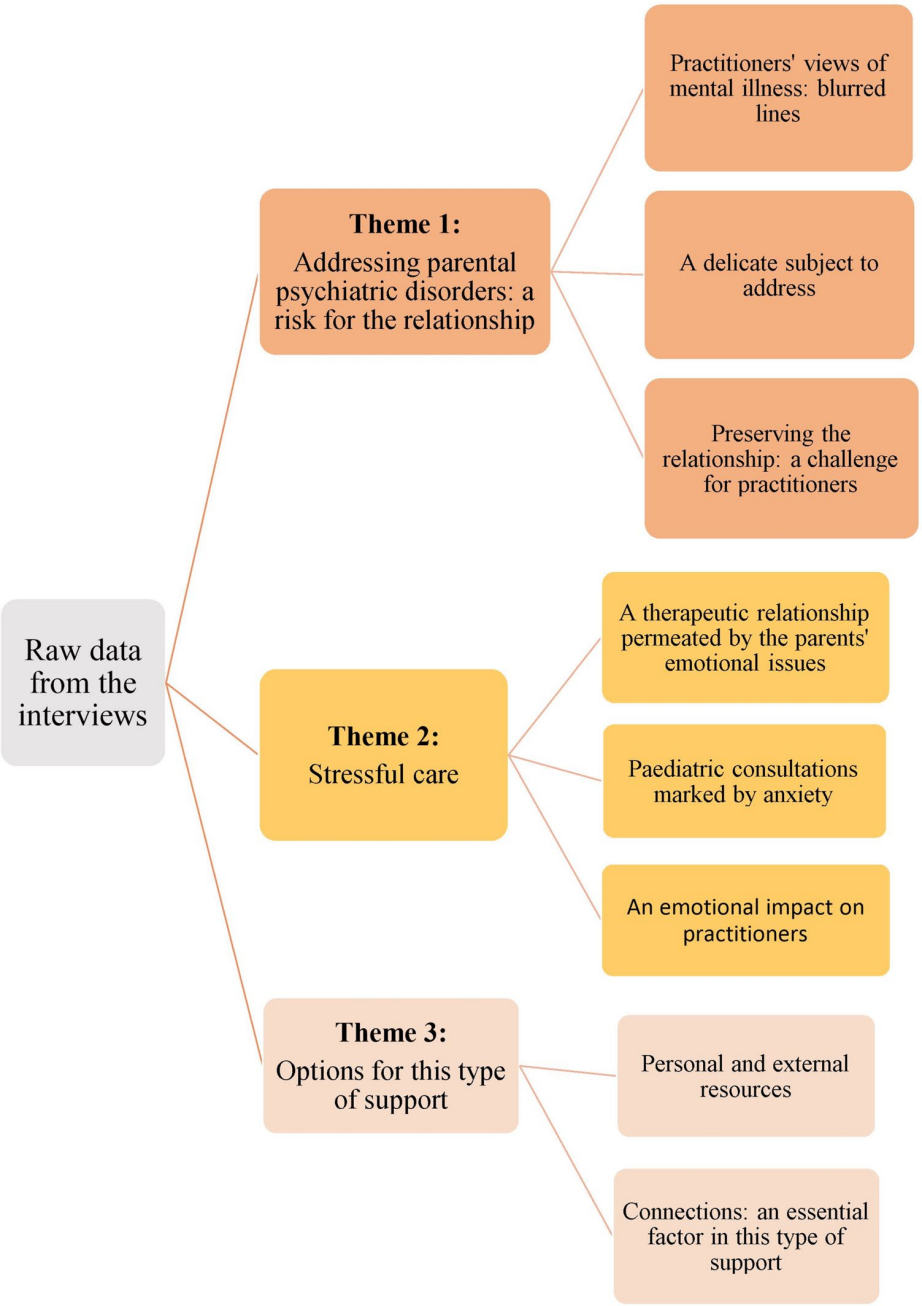
Thematic tree of results, describing the main themes and sub-themes

The results are summarised in Fig. 2.

## Discussion

This study is the first to examine the experiences of private practitioners involved in the care of children whose parents suffer from psychiatric disorders. The IPA qualitative research method is a rigorous and validated method of analysing the experience of individuals through data collected in individual interviews, which provides a better understanding of the subjects' experiences.

The physicians who were interviewed emphasised that addressing parental psychiatric disorders presents a risk to the therapeutic relationship. This notion of risk to the relationship when certain subjects are addressed has been noted in other studies. One study in which adult psychiatrists were interviewed, found that some had difficulty discussing their patients' parenting for fear of damaging the therapeutic relationship [18]. With regards to this, questioning whether so-called “anxiety-depressive” disorders fall within the field of psychiatry may be underpinned by the difficulty doctors have recognising less severe illnesses with which they themselves can identify or which they may observe in relatives, as psychiatric disorders. However, a study in Quebec of general practitioners who have more training in psychiatry, did not identify these difficulties [25]. Therefore, our study highlights the value of mental health training for primary care physicians, which is consistent with previous work [26]. Although it was not mentioned by the interviewees, using systematic questionnaires to investigate parents' mental health could also help practitioners to overcome barriers in addressing this topic.

Difficulties getting a parental psychiatric history or on-going psychiatric pathology result in late discovery of pre-existing psychiatric illnesses. However, early detection is of major importance for the prognosis of the child. Several researchers have studied the impact of preventive interventions in families with at least one parent with a mental illness [25–28]. A recent meta-analysis showed that early intervention reduced the risk of psychiatric disorders in these children by 47%, mainly through cognitive behavioural therapy or psycho-education [29]. The indirect approach to parenting difficulties by identification, used by some doctors is interesting, reminiscent of the psychotherapeutic technique of self-disclosure, which makes the doctor more accessible and normalises the patient's experience, thereby promoting the relationship [29, 30]. In parallel, the notion of a “vulnerable child syndrome" which combines risk endophenotypes and subclinical symptoms is emerging, making it possible to identify among siblings, the children most at risk for psychiatric disorders, which is the target population for these interventions [31]. These data, which contrast with the current lack of care for these high-risk children, support the development of child and adult psychiatric services to provide adequate, simultaneous and integrated follow-up of parental and child psychiatric illness from a global health perspective [31].

Practitioners are torn between not wanting to stigmatise parents and their concerns for children. Underlying this ambivalence is the fear of making mistakes and harming families. In fact, children who have parents with psychiatric disorders are at greater risk of developmental delays and psychiatric disorders compared to the general population [5, 31–36]. Moreover, the stigma of mental disorders remains strong in our societies [37–40]. These preconceptions and prejudices also apply to doctors [41], regardless of their specialities [42]. The literature is nuanced on this issue. Some researchers stress that it is not so much the psychiatric diagnosis for parents that causes a risk of negative consequences for the child, but rather the severity and chronicity. Others insist on the fact that depressed or schizophrenic parents can be excellent caregivers [43]. This uncertainty and the resulting fears are accompanied by counter-transference towards these parents, with two pitfalls: over-investment in the relationship by the physician or, on the contrary, unconscious rejection. In addition, the fear of care placement has an impact on the doctor/parent relationship, and is a source of anxiety for physicians. Yet these decisions are often necessary in the lives of these children as a form of protection or parental support. It is estimated that approximately 60–80% of parents with a severe psychiatric disorder have lost custody of at least one of their children [44]. The challenge concerning care placement is to measure the therapeutic benefit for the child, while considering the negative impact on the parent. Once again, the importance of training becomes obvious. It enables practitioners to change their approach by considering care placement as a preventive or therapeutic tool. Access to supervision or analyses of practice would be useful for practitioners in all specialities, to identify the effects of transference and counter-transference in the encounter with these parents and to avoid the pitfalls of a possible defensive distancing of parents' mental illnesses. Recent developments in practitioner attitudes have seen the emergence of programmes based on the strengths that a parent with mental illness, even a severe one, can bring to their child. For example, the psychoeducational intervention “Let’s Talk About Children” offered parents with mental illness the opportunity to address their need to feel like an effective parent, using self-regulatory processes of self-efficacy and personal agency. Indeed, becoming a parent can also be an opportunity for parents with mental illness to change their perspective on their mental illness by shifting their personal identity in the cycle of their lives, with parenthood providing feelings of pride, motivation, hope and purpose.

Physicians emphasise the support of institutions that allow them to jointly refer the parent–child dyad, and those with which they can coordinate their care through telephone links. Nevertheless, doctors describe difficulties accessing care, which stem from the insufficient resources allocated to child psychiatry. Other health professionals worldwide describe the same barriers in referring their adult patients. This observation, made in France at the national level, led to the *Assises de la Santé Mentale et de la Psychiatrie* (Mental Health and Psychiatric Conference) in September 2021, with the announcement of government commitments to remedy this situation.

On the other hand, physicians regret the lack of communication with specialists, despite the clear benefit of these connections which provide them with a better understanding of situations and help them to adapt their approach to each one, as has already been observed in other studies [23, 39]. Projects are implemented to improve such communication, such as the “CMP 2020 project” in which, through the allocation of financial resources, the CMPs of the various child psychiatry sectors of department 31 set up meeting times for partners (private doctors, maternal and child protection and child welfare agencies) to jointly reflect on and co-construct a partnership based on complex situations. To address their isolation, some practitioners suggest the creation of a multidisciplinary network of health professionals aimed at extending support for these follow-ups by providing advice and management guidance. Multidisciplinary and integrated mental health care that targets youth, leads to an improvement in their psychological status through a wide range of available interventions, and also has a demonstrated medico-economic impact [45–49].

This desire to work in partnership is part of a will to improve professional practices, but also the well-being of practitioners who, as we have seen, are confronted with stressful follow-ups, sources of identification, and sometimes of psychological suffering.

This research has several limitations. The participants were recruited from only one French department. A selection bias can also be mentioned due to the voluntary nature of the recruitment. The physicians who participated in the study probably had a particular interest in the subject. Finally, as this study was conducted within the French health care system, these results are not directly generalisable to early childhood care systems abroad.

## Conclusion

Our study provides a better understanding of the experiences of early childhood physicians in caring for children whose parents suffer from psychiatric disorders. We highlighted a constant fear of a breakdown in the therapeutic relationship with the parent in this joint care. Physicians appear to be ambivalent about caring for these children, wavering between their concerns about the parent–child bond and their desire not to stigmatise these families. The emotional burden linked to this type of care is significant and the practitioners interviewed mentioned their isolation during follow-up, as well as a lack of time for communication. Our research also suggests that strengthening multidisciplinary work by promoting inter-professional connections would be beneficial to the care of these families with complex needs, and essential to improve the experience of these physicians.

Professional training in child and adult psychiatric illnesses and child protection would help to combat physicians' fear of confronting mental illness and would contribute to reducing delays in the implementation of preventive actions which are likely to improve the developmental prognosis of these children with parents who suffer from psychiatric disorders.

## Data Availability

All data, audio recordings and analyses from the research are available on request.
